# Types of health service utilization in Mumbai slums: a community-based survey

**DOI:** 10.1186/s13104-023-06557-y

**Published:** 2023-10-24

**Authors:** Maitreyee Patwardhan, Shang-Ju Li, Thomas Miles, Harshwardhan Dere, Dipika Khushalani, Shripad Desai

**Affiliations:** 1 Americares India Foundation, Mumbai, India; 2 Americares Stamford, Stamford, USA

**Keywords:** Health care, Access, Utilization, Slums, India, Mumbai, Behaviors, Health seeking

## Abstract

**Objective:**

Sociodemographic factors play a crucial role in shaping the health-seeking behaviors of individuals residing in slum areas, particularly in their choice of healthcare facilities. Recognizing the importance of strengthening the existing healthcare systems, this research project was undertaken with the primary objective of comprehending the health-seeking behaviors among residents of Mumbai’s slum dwellings in India. To achieve this goal, a comprehensive cross-sectional community needs assessment was conducted spanning from October 2018 to January 2019.

**Results:**

432 respondents reported utilizing at least one health facility in the past year. They reported using private hospitals (172), public hospitals (208), Community Health clinics [23], or other healthcare services (29). To gain further insights into the factors influencing these choices, logistic regression analysis was conducted. The analysis revealed that being female was found to be negatively associated with the selection of a general practitioner as a preferred healthcare provider. On the other hand, higher levels of education and income were found to have a positive association with the preference for private hospitals. Conversely, these factors were negatively associated with the choice of government hospitals.

**Supplementary Information:**

The online version contains supplementary material available at 10.1186/s13104-023-06557-y.

## Introduction

An estimated 41.8% of Mumbai’s population lives in urban slums [1]. Slum health is dictated by a high degree of poverty, low access to resources combined with poor living conditions [2], which leads to a greater degree of infections, disease, and other health conditions. Thus, the need for quality healthcare remains high. Healthcare in India is decentralized, with the state and federal governments responsible for policy and provision [3]. The Directorates of Health Services and the Departments of Health and Family Welfare oversee healthcare at the state level [4]. The country has a mixed healthcare system, with approximately 10% of hospitals being government-run, and the rest operated by private for-profit sectors or charitable organizations [3, 4]. The preference for accessing public healthcare due to lower cost has been observed in Mumbai, but barriers such as long wait times, low quality of care, and accessibility remain [5, 6]. In the Panjrapole slum of Mumbai, 31% of respondents consulted private doctors due to dissatisfaction with the quality of care at government hospitals [2]. This has led to a significant growth in the number of private sector hospitals throughout the country, largely due to perceived poor-quality care in public facilities and the rapid expansion of medical tourism [3].

Sociodemographic factors impact healthcare access in India, including age, education, religion, family size, residential community, and availability of basic needs [7, 8]. Women face gender inequality in healthcare access, reporting lower utilization and satisfaction [9]. Education, employment of women, and family type are also significant determinants of health care utilization in urban slums [10]. While health systems in urban slums remain inadequate, Americares India Foundation (AIF) operates seven mobile health centers (MHCs) that provide free medical services to patients in 130 communities every two weeks. AIF’s electronic medical records show that respondents are relatively young, highly educated, mostly Hindu, and live in nuclear families with access to basic needs. To improve patient access to comprehensive clinical services, AIF commissioned a cross-sectional, quantitative study to understand the primary and specialty care provider landscape and health-seeking behaviors in Mumbai’s slums. Our study questions were:

1. What are the sociodemographic factors of slum community residents that predispose their health-seeking behaviors?
2. What are the barriers and resources existing in local slum communities in Mumbai?

## Methods

Ipsos Group S.A., a social research organization with Identification Number (CIN) U74130MH2004PTC146904 and registration number 146,904, was contracted in 2018 to develop a landscape study assessing health seeking behaviors among community residents of Mumbai, focused on their choices around the type of health facility. Power analyses determined that 2,600 households were ideal for quantitative analysis. Households were randomly selected across 13 wards in Mumbai. In each ward, four sites at which Americares MHC had active services were randomly selected. At each site, interviewers walked in two different directions, conducting door-to-door sequential surveying. Within each selected household, one adult was selected randomly for an interview. Surveying continued until the desired sample size (n = 2,862 households) was reached.

To answer our study questions, demographic characteristics such as age, gender, education, and occupation, as well as household profiles and health-seeking behaviors were collected as independent variables (Supplementary Material 1). Participants were asked about their awareness, availability, affordability, and accessibility of health facilities in the area, and their perceptions about common health problems. The questionnaire included a nested survey question to assess the type of provider used, with response options including government and private hospitals, community health centers, and private practitioners. Responses were recoded into binary measures to indicate whether the facility was accessed or not. Ayurvedic, nursing home, private clinic, and private doctor options were omitted from final analyses due to low sample sizes. Prior to administering the structured questionnaire to a larger population, pilot testing was conducted to ensure that the questions were clear, concise, and easy to understand. Based on the feedback received, necessary adjustments were made to ensure that the final version of the survey accurately captured the intended information.

Data analysis was conducted using STATA/MP version 15 for Mac (32) [11]. ​​​​Logistic regression modeling was used to examine the association of sociodemographic factors - gender, age, marital status, education, and income level - with each outcome (binary measures of accessing government hospital, private hospital, community health center or private practitioner). Each of the analyses show how the sociodemographic factors affect the choice of the specific healthcare facility as represented by a odds ratio. P-values of less than 0.05 were considered statistically significant.

## Results

Among all respondents (N = 2862), 15% (n = 432) reported using any type of health facility within the past year. community health clinics (n = 23, 5.3%) and “other form of healthcare service” (n = 29, 6.7%) have relatively smaller sample sizes and were excluded in the following analysis. 380 respondents reported using public or private facilities, 45.3% (n = 172) reporting use of private hospitals, and 54.7% (n = 208) reported use of public hospitals. 52.9% (n = 201) were male and 60/8% (n = 231) were female. The mean age of the participants reporting use of a healthcare facility in the past year was 30.45 (Table 1). Among these participants, the majority were female, reported being in a relationship, and had at least a secondary education. Most (81.1%) reported having a household monthly income greater than 10,000 rupees per month (~ 130 USD). In bivariate test of association, the choice of healthcare facility type within the past year was found to be associated with sex of the respondent, civil/marital status, and education; however, the choice of healthcare facility type was not significantly associated with reported household income.

**Table 1:** Descriptive characteristics of individuals visiting health facilities in the past year

	Type of health facility visited in the past year N (%)			
Characteristic	Private hospital	Public hospital	Total	P-value
Male	86 (50.0)	88 (42.3)	174 (45.8)	< 0.001
Female	86 (50.0)	120 (57.7)	206 (54.2)	
In a relationship (Married, Living together)	100 (58.1)	134 (64.4)	234 (61.6)	0.013
Not in a relationship (Single, Divorced, Widow, Separated)	72 (41.9)	74 (35.6)	146 (38.4)	
Primary education or less	54 (31.4)	93 (44.7)	147 (38.7)	0.015
Secondary education or more	118 (68.6)	114 (54.8)	232 (61.3)	
Don’t know / No response	0 (0.0)	1 (0.5)	1 (0.0)	
Less than 5,000 INR	4 (2.3)	8 (3.8)	12 (3.2)	0.224
5,001–10,000 INR	25 (14.5)	52 (25.0)	77 (20.2)	
10,001–15,000 INR	50 (39.1)	70 (33.7)	120 (31.6)	
15,001–20,000 INR	35 (20.4)	33 (15.9)	68 (17.9)	
More than 20,000 INR	48 (27.9)	35 (16.8)	83 (21.9)	
Don’t know / Can’t say	10 (5.8)	10 (4.8)	20 (5.3)	
Mean age (Mean/SD)	30.6 (11.7)	31.3 (10.8)	30.5 (11.0)	0.036

Multivariable analysis considered the effects of respondent sex, civil/marital status, level of education, and reported household average monthly income on utilization of health care facilities. Table 2 showed that being female was significantly negatively associated with choosing a general practitioner (odds ratio: 0.80 CI: 0.67–0.92). When choosing a private hospital, higher education (odds ratio: 1.17 CI: 1.00–1.37) and higher income (odds ratio: 1.32 CI: 1.09–1.60) are significantly positively associated. High income was found to be significantly negatively associated with choosing a government hospital (odds ratio: 0.79 CI: 0.66–0.96).

**Table 2:** Regression models of sociodemographic covariates on type of service provider accessed

Characteristic	General Practitioner				Private Hospital			Government Hospital		
	OR	Lower CI	Upper CI	OR		Lower CI	Upper CI	OR	Lower CI	Upper CI
Intercept	1494.71	0.00	Inf	0.10		0.03	0.39	1.93	0.52	7.08
Female	0.80**	0.67	0.92	1.02		0.99	1.04	1.00	0.98	1.02
Age	0.00	-Inf	Inf	0.90		0.45	1.64	1.27	0.70	2.57
Married	894.74	0.00	Inf	0.97		0.50	2.04	0.97	0.46	1.85
Education	1.14	0.75	1.73	1.17*		1.00	1.37	0.91	0.78	1.06
Income	0.90	0.56	1.44	1.32**		1.09	1.60	0.79*	0.66	0.96

* p < 0.05

** p < 0.01

## Discussion

This study analyzed the health-seeking behaviors of Mumbai’s slum residents and identified key factors that influence their choice of health facility. The study drew on a landscape survey conducted by AIF from October 2018 to January 2019, which supported previously published findings on health care service utilization in urban slums. The study found that women were less likely to seek care from private hospitals, which aligns with prior research indicating that health care expenditure is generally lower for women in India compared to men [8]. Additionally, older women were found to be more likely than older men to access public rather than private health care [12].

This study revealed a positive association between higher income and education and the use of private hospitals for seeking healthcare, in line with existing literature [10, 13]. However, civil/marital status did not appear to significantly impact the choice of healthcare facility. Other studies have suggested that married individuals are more likely to prefer private healthcare compared to widowed individuals [12]. Despite many mothers having antenatal care visits in public healthcare facilities, almost 50% of the mothers in this study visited private healthcare facilities for antenatal care [10]. This disparity suggests a possible distrust among women regarding the quality of services provided in public healthcare facilities and highlights the need for further research on health-seeking behaviors.

This study revealed a positive association between high income and the use of private hospitals, and a negative association with government hospitals, when compared to low-income families. According to literature, low-income families in Mumbai tend to prefer publicly run Brihanmumbai Municipal Corporation (BMC) hospitals [14]. Previous studies have found that patients were dissatisfied with the lack of necessary medical supplies available at government facilities, which often resulted in bearing the cost of treatment out of pocket or making informal payments to receive care [4, 6]. Our findings also align with international studies. Multiple studies have found a correlation between health care seeking behavior and socioeconomic indicators. These studies suggest that individuals with higher levels of education and income are more likely to choose private hospitals over public hospitals [15]. Additionally, gender disparities have been observed, with women being less likely to choose a general practitioner compared to men [16].

The literature strongly supports the association between educational and sociodemographic factors and health seeking behaviors among slum dwellers in India. Studies have shown that inadequate health seeking behavior is a factor in up to 70% of child deaths [6]. Despite the free public healthcare system in India, public facilities are underutilized, possibly due to the reported poor quality of care. This study’s findings on health seeking behaviors for different types of facilities align with previous research, highlighting the need for improved monitoring and evaluation of healthcare services for urban slum populations. Overall, this study contributes to the extensive literature on healthcare utilization in India [17–19].

## Conclusions

This study examines how sociodemographic indicators affect the use of healthcare facilities in urban areas, with a focus on identifying factors that improve access to affordable and impactful health services for underserved populations. Factors such as cost, distance, knowledge of available services, and the need for specialized care were found to be crucial considerations when selecting a healthcare facility. Despite the availability of a large public healthcare network, many residents of slum areas still opted for private providers, particularly those with higher income and education levels. To address this challenge, the study mapped primary and specialty care providers in Mumbai and worked to enhance patient access to comprehensive clinical services [20, 21].

## Limitations

This study aimed to understand the healthcare provider landscape in Mumbai and improve patient access to comprehensive clinical services. However, due to the small sample size of only 432 respondents who had visited a health facility in the past year, the analyses were constrained and depowered. This was further exacerbated by small sub sample sizes of those who utilized community health centers or other forms of healthcare (n = 23 and n = 29, respectively). The use of self-reported measures introduced response biases such as social desirability and recall, and demographic characteristics alone cannot provide a complete picture of an individual’s healthcare needs [22]. The complex and multifaceted relationship between sociodemographic factors and healthcare utilization, including unique barriers and challenges, requires future studies to include more objective measures and increase sample sizes for sub sample analyses [23, 24].

### Electronic supplementary material

Below is the link to the electronic supplementary material.

Supplementary Material 1: Completed survey

## Data Availability

The datasets used and/or analyzed during the current study are available from the corresponding author on reasonable request.

## Abbreviations

AIF: Americares India Foundation
ANC: Antenatal Care
BMC: Brihanmumbai Municipal Corporation
CAPI: Computer Assisted Personal Interview
MHC: Mobile Health Centers
RIST: Rural India Supporting Trust
SE: Standard Error
USD: United States Dollar
