# Supplementary material for: Types of health service utilization in Mumbai slums: a community-based survey
Source: BMC Res Notes. 2023 Oct 24;16:289. doi: 10.1186/s13104-023-06557-y (PMC10598993; doi:10.1186/s13104-023-06557-y)
Supplement: Supplementary file 1 — Supplementary Material 1: Completed survey [file 13104_2023_6557_MOESM1_ESM.doc]

|  |
| --- |
| Community Needs Landscape Study |
| Mumbai, India |

| 4-1-2018 |
| --- |

**Interviewer Instruction:** Please contact AN ADULT MEMBER OF THE HOUSEHOLD (PREFERABLY FEMALE OR ONE WHO IS MOST INFORMED OF THE FAMILY HEALTH STATUS AND HEALTH SEEKING BEHAVIOUR) for interview.

If not available, then contact the second-most knowledgeable person about household member details; if not available, then House should be revisited later (the time of day being different to the time that the first visit is made; inquire from other members at what time the relevant person will be home).

**इन्टरव्यूअर के लिए सुचना :** इन्टरव्यू के लिए कृपया घर के किसी बड़े सदस्य से बात करे (मुख्यतः किसी महिला से या उससे जो परिवार की स्वास्थ्य स्थिति और स्वास्थ्य गतिविधियों के बारेमे सबसे अधिक जानकारी रखते है).

यदि वह व्यक्ति उपलब्ध नहीं हो तो दुसरे उस व्यक्ति से बात करे जिन्हें घर के सदस्यों के बारेमे विस्तार में जानकारी हो, यदि वह व्यक्ति उपलब्ध नहीं हो तो उस घर को बाद में आकर मिले (जिस दिन बाद में आकर मुलाकात की जाएगी वह दिन समय पहले इस दिन के समय से अलग होना चाहिए, घर के अन्य सदस्यों से जानकारी ले की वह उचित व्यक्ति किस समय घर पर उपस्थित होंगें).

**Interviewer to read:** We are conducting a survey to understand awareness of community about various issues that affect your living and how you deal with the issues. This research is being conducted by Americares an NGO that works in Mumbai and IPSOS Research, a research organization. We would like to ask you some questions that will help us to understand the health needs of families in your area and how services can be improved to meet your needs. The survey will take less than an hour, and your name is not tied to your responses – therefore, you will be answering this survey anonymously. Are you willing to participate?

**इन्टरव्यूअर पढ़े :** आपके जीवन को प्रभावित करने वाली विभिन्न समस्याएँ और आप उन समस्यों से किस प्रकार निपटते है इसकी जागरूकता समाज में कितनी है यह समझने के लिए एक सर्वे आयोजित कर रहे हैं. यह सर्वे अमेरिकेयर्स जो एक एनजीओ है और इप्सोस रिसर्च जो एक रिसर्च ऑर्गेनाइजेशन है द्वारा आयोजित किया जा रहा है. हम आपसे कुछ प्रश्न पूछना चाहते हैं जिससे आपके क्षेत्र में परिवारों की स्वास्थ्य आवश्यकताओं को समझने के लिए और आपकी आवश्यकताओं को पूरा करने के लिए सेवाओं में कैसे सुधार किया जा सकता है यह समजने में हमें सहायता होगी. इस सर्वे में एक घंटे से भी कम का समय लगेगा और आपके जवाबों के साथ आपका नाम नहीं जोड़ा जाएगा तो इसका मतलब होता है की आप इस सर्वे में गुमनाम रूप से जवाब देंगें. क्या आप सहभाग लेना चाहतें है?

| **Q No** | **Question** | **Responses** | **Code** |
| --- | --- | --- | --- |
| A1 | In which language you would be comfortable in answering this survey?  कौन सी भाषा में आप इस सर्वे का उत्तर देने के लिए सुविधाजनक महसूस करेंगे? | Hindi | 1 |
|  |  | Marathi | 2 |
| A2 | City | Mumbai |  |
| A3 | Ward Number |  |  |
| A4 | How long you are living in this community / area  आप इस मोहल्ले/ इलाके में कब से रह रहे हैं?  **Interviewer instruction: If less than 1-year mention ‘0’**  इंटरव्यूअर को निर्देश : यदि 1 वर्ष से कम है तो ‘0’ कहें | Record the response in years ______  वषों में उत्तर को रिकॉर्ड करें |  |
| A5 | Community |  |  |
| A6 f | Household Number |  |  |
| A6 g | Name of respondent |  |  |
| A6i | Landmark |  |  |
| A6j | Address |  |  |
| A6h | Investigator Name |  |  |
| A8 | Investigator ID  ENTER YOUR MOBILE NUMBER |  |  |
| A8a | Supervisor Name |  |  |
| A12 | GPS coordinates |  |  |
| A13 | Device Id |  |  |
| A15 | Unique Id of Device (IMEI) |  |  |
|  | |  | |
| A17 | Interview status  **Interviewer to record** | Incomplete | 0 |
|  |  | Complete | 1 |
|  |  | Discarded | 2 |
|  |  | HoH not available after multiple visits | 3 |
|  |  | HoH refused to participate | 4 |
|  |  | Individual refused to participate | 5 |
|  |  | Individual not available after multiple visits | 6 |
|  |  | Household locked after multiple visits | 7 |

| **SECTION -1 : RESPONDENT PROFILE** |
| --- |

1. **Note down the sex of the respondent. OBSERVE AND CODE. DON’T ASK**

**रिस्पोंड़ेन्ट महिला है या पुरुष यह नीचें लिखे.**

**SINGLE CODING ONLY**

| Male पुरुष | 1 |
| --- | --- |
| Female महिला | 2 |

1. **Please tell me your age? RECORD THE ANSWER IN THE BOX BELOW**

**कृपया हमें आपकी आयु बताएँ?**

| ______________________________________ YEARS  ___________________________________ वर्ष |
| --- |

**POST CODE THE AGE IN THE GRID BELOW**

**Scripter instruction: Terminate if coded 1 or 2 or 3**

| 0 – 5 years 0 – 5 वर्ष | 1 |
| --- | --- |
| 6 -14 years 6 -14 वर्ष | 2 |
| 15 – 17 years 15 – 17 वर्ष | 3 |
| 18 – 24 years 18 – 24 वर्ष | 4 |
| 25 -40 yearsc 25 - 40 वर्ष | 5 |
| 41-49 years 41-49 वर्ष | 6 |
| 50-60 years 50-60 वर्ष | 7 |
| 60+ years 60+ वर्ष | 8 |

1. **What is highest level of education/schooling you have received? SINGLE CODING ONLY**

आपने उच्चतम् शिक्षा कहा तक प्राप्त की है?

| Illiterate  निरक्षर | 1 |
| --- | --- |
| Educated up to 5th standard  कक्षा 5 तक शिक्षित | 2 |
| Educated up to 8th standard  कक्षा 8 तक शिक्षित | 3 |
| Secondary up to class 10  कक्षा 10 तक माध्यमिक | 4 |
| Senior Secondary up to class 12  12 वीं कक्षा के वरिष्ठ माध्यमिक | 5 |
| Graduate  ग्रेजुएट | 6 |
| Post Graduate  पोस्ट ग्रेजुएट | 7 |
| Diploma/ITI  डिप्लोमा/आयटीआय | 8 |
| Vocational Courses  व्यवसायिक कोर्सेस | 9 |
| Any other (please specify)  कोई अन्य (कृपया स्पष्ट करे) |  |
| Don't Know/Can't Say  मालूम नहीं / कह नहीं सकते | 98 |

1. **Which caste do you belong to? (Prompt, if you are comfortable answering) SINGLE CODING ONLY**

**आप किस वर्ग से संबंधित हैं**?

| General  जनरल | 1 |
| --- | --- |
| Scheduled Caste  अनुसूचित जाति | 2 |
| Scheduled Tribe  अनुसूचित जनजाति | 3 |
| Other Backward Classes  अन्य पिछड़ा वर्ग | 4 |
| Don’t Know  पता नहीं | 98 |
| Refused to answer  उत्तर देने से मना कर दिया | 99 |

1. **Which religion do you practice? SINGLE CODING ONLY**

**आप किस धर्म की पालन करते है?**

| Hindu  हिंदू | 1 |
| --- | --- |
| Muslim  मुस्लिम | 2 |
| Christian  क्रिस्चन/ईसाई | 3 |
| Sikh  सिख | 4 |
| Buddhist/Neo-Buddhist  बौद्ध / नव-बौद्ध | 5 |
| Jain  जैन | 6 |
| Jewish  यहूदी | 7 |
| Parsi/Zoroastrian  पारसी | 8 |
| No religion  कोई धर्म नहीं | 9 |
| Other (please specify)  अन्य (कृपया स्पष्ट करे) |  |
| Don’t Know  पता नहीं | 98 |
| Refused to answer  उत्तर देने से मना कर दिया | 99 |

| **SECTION -2: DETAILS OF THE FAMILY** |
| --- |

1. **What is the type of family you are living in? SINGLE CODING ONLY**

**आप किस प्रकार के परिवार रह रहे है?**

| Nuclear Family  एकल परिवार | 1 |
| --- | --- |
| Joint Family  संयुक्त परिवार | 2 |

1. **How many members are there in your household, in the following age – group:**

**आपके घर में निम्मलिखित आयु-समूह के कितने सदस्य रहते है :**

**WRITE IN NUMERALS
ALLOW NUMERALS BETWEEN 1-10**

| **Age group** | **Males**  **पुरुष** | **Females**  **महिला** |
| --- | --- | --- |
| 0 – 5 years  0-5 वर्ष |  |  |
| 6 -14 years  6-14 वर्ष |  |  |
| 15 – 17 years  15-17 वर्ष |  |  |
| 18-24 years  18-24 वर्ष |  |  |
| 25 -40 years  25-40 वर्ष |  |  |
| 41-49 years  41-49 वर्ष |  |  |
| 50-60 years  50-60 वर्ष |  |  |
| 60+ years  60+ वर्ष |  |  |

1. **Who is the head of the household??**

**आपके घर के मुखिया कौन है?**

**INTERVIEWER TO READ:**

**इन्टरव्यूअर पढ़े :**

**BY HEAD OF HOUSEHOLD, I MEAN THE PERSON WHO TAKES IMPORTANT DECISIONS IN THE HOME and/or has major role in taking family level decisions**

**घर के मुखिया से मेरा मतलब है, वह व्यक्ति जो घर के महत्वपूर्ण निर्णय लेता हो और/ या पारिवारिक स्तर के निर्णय लेने में जिसकी मुख्य भूमिका हो**

**SCRIPTING INSTRUCTION: MULTIPLE CODING POSSIBLE**

| Myself  खुद | 1 |
| --- | --- |
| Father  पीताजी | 2 |
| Mother  माँ | 3 |
| Brother  भाई | 4 |
| Sister  बहन | 5 |
| Husband  पति | 6 |
| Wife  पत्नी | 7 |
| Father-in-law  ससुर | 8 |
| Mother-in law  सांस | 9 |
| Son-in-law  जमाई | 10 |
| Daughter-in-law  बहूँ | 11 |
| Others-Specify  अन्य – स्पष्ट करे |  |

1. **What is the main occupation of the head of the household?**

**आपके घर के मुखिया का मुख्य व्यवसाय क्या है?**

**SCRIPTING INSTRUCTION:**

**SINGLE CODING**

| Self employed Professional  स्वनियोजित व्यवसायिक | 1 |
| --- | --- |
| Salaried – Government service  वेतनकर्मी- सरकारी नौकरी | 2 |
| Salaried – Private company / service  वेतनकर्मी- निजी कंपनी/ नौकरी | 3 |
| Petty Trade  छोटा व्यापार | 4 |
| Buisness  व्यवसाय | 5 |
| Skilled worker  कुशल मजदूर | 6 |
| Casual Labourer  अनौपचारिक मजदूर | 7 |
| Security guards  सिक्योरिटी गार्ड | 8 |
| Unemployed  बेरोजगार | 9 |
| House wife  गृहिणी | 10 |
| Others (Specify)  अन्य (स्पष्ट करे) | 11 |

**9b. Apart from Head of the household how many members in the family are involved in any economic activity that generates income for them?**

**आपके घर के मुखिया को छोड़कर परिवार के कितने सदस्य कोई भी आर्थिक गतिविधि में शामिल हैं जो उनके लिए आय उत्पन्न करती है?**

**Scripter instruction: have logic check with Q7**

**IF CODED**

**स्क्रिप्टर को निर्देश: Q7 के साथ लॉजिक चेक करें यदि कोडेड है**

| Number of family members involved in economic activity  **आर्थिक गतिविधि में शामिल परिवार के सदस्यों की संख्या** | |  |  | | --- | --- | |
| --- | --- | --- | --- |
| Only head of the household is involved in the economic activity  आर्थिक गतिविधि में केवल मुखिया शामिल हैं | **99** |

**9c. What are their occupations?**

**उनके व्यवसाय क्या हैं?**

**SCRIPTING INSTRUCTION: MULTIPLE CODING POSSIBLE**

**स्क्रिप्टर को निर्देश: एक से अधिक कोडिंग संभव**

| Self employed Professional  स्वनियोजित व्यवसायिक | 1 |
| --- | --- |
| Salaried – Government service  वेतनकर्मी- सरकारी नौकरी | 2 |
| Salaried – Private company / service  वेतनकर्मी- निजी कंपनी/ नौकरी | 3 |
| Petty Trade  छोटा व्यापार | 4 |
| Buisness  व्यवसाय | 5 |
| Skilled worker  कुशल मजदूर | 6 |
| Casual Labourer  अनौपचारिक मजदूर | 7 |
| Security guards  सिक्योरिटी गार्ड | 8 |
| Unemployed  बेरोजगार | 9 |
| House wife  गृहिणी | 10 |
| Others (Specify)  अन्य (स्पष्ट करे) | 11 |

1. **What is the average monthly income of your household, combined of all wage earners?**

**SINGLE CODING ONLY**

आपके घर के सभी कमानेवाले सदस्यों की मिलाकर कुल मासिक आय कितनी है?

| Less than 5,000  5000 से कम | 1 |
| --- | --- |
| 5001-10,000 | 2 |
| 10,001-15,000 | 3 |
| 15,001-20,000 | 4 |
| More than 20,000  20000 से अधिक | 5 |
| Don’t Know/Can’t Say  मालूम नहीं / कह नहीं सकते | 99 |

| **SECTION -3: ASSET INDEX OF THE FAMILY** |
| --- |

1. **A.** For heating, lighting, or cooking does your household use? **MULTIPLE CODING POSSIBLE**

गरम करने के ले, जलाने के लिए या खाना पकाने के लिए क्या आपके घर में इसका इस्तमाल होता है?

|  | **Yes**  **हाँ** | **No**  **नहीं** |
| --- | --- | --- |
| Kerosene  केरोसिन |  |  |
| Firewood/twigs  लकड़ी / टहनियाँ |  |  |
| LPG  एलपीजी |  |  |

**11 B.** Does your household have? **MULTIPLE CODING POSSIBLE**

क्या आपके घर में यह है?

| Household Items  घर का सामान |  |  |
| --- | --- | --- |
| A colour T.V.  कलर टिव्ही | 1 | 2 |
| Cable / dish T.V.  केबल / डिश टिव्ही | 1 | 2 |
| Pressure Cooker  प्रेशर कुकर | 1 | 2 |
| Refrigerator  रेफ्रीजरेटर | 1 | 2 |
| Mixer / Grinder  मिक्सर/ग्राइंडर | 1 | 2 |
| Air Cooler  एयर कूलर | 1 | 2 |
| Motorcycle/Scooter  मोटर साइकिल / स्कूटर | 1 | 2 |
| Toilet inside household  घर के अंदर टॉयलेट | 1 | 2 |

1. **Where do the members of your household go for defecation? SINGLE CODING ONLY**

**आपके घर के सदस्य शौच के लिए कहाँ जाते है?**

| Sulabh Shauchalya  सुलभ शौचालय | 1 |
| --- | --- |
| Open defecation  खुले में शौच | 2 |
| Flush latrine  फ्लश शौचालय | 3 |
| No response  कोई उत्तर नहीं | 99 |

| **SECTION -4: WATER AND SANITATION** |
| --- |

1. **What is the main source of drinking water for the household?**

**आपके घर के पीने के पानी का मुख्य स्त्रोत क्या है?**

**MULTIPLE CODING ONLY**

| **Piped water पाइप का पानी** | |
| --- | --- |
| Piped into dwelling  घर में पाइप | 1 |
| Piped to yard / plot  चौक/प्लाट में पाइप | 2 |
| Piped to neighbour  पडोसी का पाइप | 3 |
| Public tap / standpipe  सार्वजनिक नल / स्टैंडपाइप | 4 |
| Tube well / borehole  ट्यूब वेल / बोरहोल | 5 |
| Filtration plant  निस्पंदन संयंत्र | 6 |
| **Dug well खोदा हुआ कुआं** | |
| Protected Well  संरक्षित कुआं | 7 |
| Unprotected Well  असुरक्षित कुआं | 8 |
| **Spring** ज़राना | |
| Protected Spring  संरक्षित ज़राना | 9 |
| Unprotected Spring  असुरक्षित ज़राना | 10 |
| Tanker Truck  टैंकर ट्रक | 11 |
| Cart with Small Tank  गाड़ी के साथ छोटा टैंक | 12 |
| Surface water (river, dam, lake, pond, stream, canal, irrigation channel)  भूतल का पानी (नदी, बांध, झील, तालाब, धारा, नहर, सिंचाई चैनल) | 13 |
| **PACKAGED WATER पैक किया हुआ पानी** | |
| Bottled Water  बोतल बंद पानी | 14 |
| Others (please specify)  अन्य (कृपया स्पष्ट करे) | 15 |
| **Others** अन्य | |
| Rainwater  बारिश का पानी | 16 |

13 b. Where is the drinking water storage container kept?

पीने के पानी कस स्टोरेज कंटेनर कहाँ रखा गया है?

Investigator instruction: Observe if possible, otherwise, ask respondent

जाँच करने वाले को निर्देश: निरीक्षण करें यदि संभव है, अन्यथा रिस्पॉन्डंट से पूछें

Scripter instruction: If coded 4 skip to Q14

स्क्रिप्टर को निर्देश: यदि 4 कोडेड है तो छोड़कर Q14 पर जाएँ

| At a height and out of reach of small children and pets  ऊँचाई पर है और छोटे बच्चों और पालतू जानवरों के पहुँच से बाहर है | 1 |
| --- | --- |
| At a height but within reach of small children and pets  ऊँचाई पर है लेकिन छोटे बच्चों और पालतू जानवरों के पहुँच में है | 2 |
| On the ground  ज़मीन पर है | 3 |
| Do not store water at home  घर पर पानी जमा नहीं करते | 4 |

13 c. Is the drinking water container kept covered?

क्या पीने वाले पानी का कंटेनर ढक कर रखा हुआ है?

**Investigator instruction: Observe if possible, otherwise, ask respondent**

जाँच करने वाले को निर्देश: निरीक्षण करें यदि संभव है, अन्यथा रिस्पॉन्डंट से पूछें

| Yes  हाँ | 1 |
| --- | --- |
| No  नहीं | 2 |

13 d. How is drinking water taken out of the storage?

स्टोरेज से पीने वाले पानी को कैसे निकाला जाता है?

**Interviewer instruction – if possible please request a glass of water from respondent and observe how it is taken out, otherwise, ask respondent**

**इंटरव्यूवर को निर्देश: यदि संभव है तो कृपया रिस्पॉन्डंट से एक ग्लास पानी का निवेदन करें और निरीक्षण करें कि उसे कैसे लिया जाता है अन्यथा रिस्पॉन्डंट से पूछें**

| By pouring / tilting  उंडेलकर/ झुका कर | 1 |
| --- | --- |
| Through tap in vessel  बरतन के नल से | 2 |
| Use mug/cup with long handle / long handled ladle  मग का इस्तेमाल करते हैं/ लम्बे हैंडल वाला कप/ लम्बे हैंडल का चमचा | 3 |
| Dip mug/cup  डुबोने वाला मग/ कप | 4 |
| Taken Water from a tap  नल से पानी लिया | 5 |
| Others specify ___________  अन्य बताएं____________ | 6 |

1. **How do you make the water safe for drinking? MULTIPLE CODING ONLY**

**पीने के पानी को आप किस प्रकार से सुरक्षित बनाते है?**

| Boil  उबालकर | 1 |
| --- | --- |
| Use alum  फिटकरी का प्रयोग | 2 |
| Add bleach/chlorine tablets or liquid chlorine  ब्लीच / क्लोरीन गोलियां या तरल क्लोरीन को मिलाते है | 3 |
| Strain through a cloth  कपडें से छानते है | 4 |
| Use water filter (ceramic/ sand/composite/etc.)  वॉटर फिल्टर का प्रयोग करते है (सिरेमिक (चीनी मिट्टी)/ रेत / समग्र / आदि) का प्रयोग करते है | 5 |
| Any other (please specify)  अन्य (कृपया स्पष्ट करे) |  |
| Do not purify water at all  पानी जरा भी शुद्ध नहीं करते है | 99 |

| **SECTION -4: DISEASE BURDEN IN THE COMMUNITY** |
| --- |

1. **What are the most common health problems / diseases in your area / among neighbours (इलाके में/ पड़ोसियों के बीच में)?**

(Those problems which have the greatest impact on overall community health)

**आपके मोहल्ला(एरिया) में सबसे आम स्वास्थ्य समस्याएं / बीमारियां क्या हैं?** (वह समस्याएं जिनका समग्र मोहल्ला(एरिया) स्वास्थ्य पर सबसे बड़ा असर पड़ता है)

**Probe – Multiple options**

| Malaria | मलेरिया | 1 |
| --- | --- | --- |
| Dengue | डेंगू | 2 |
| Japanese Encephalitis (JE) | जापानी एन्सेफलाइटिस (जेई) | 3 |
| Chikungunya | चिकनगुनिया | 4 |
| Filaria | फाइलेरिया | 5 |
| Hookworm Infection | पेट का इन्फेक्शन | 6 |
| Influenza | इंफ्लुएंजा/ज़ुकाम | 7 |
| Jaundice | पीलिया | 8 |
| Tuberculosis | टीबी | 9 |
| Diarrhoeal Diseases | अतिसार रोग(लूज मोशन/दस्त) | 10 |
| Typhoid | आंत्र ज्वर/टायफ़ायड | 11 |
| Hepatitis | हेपेटाइटिस/जिगर में सूजन | 12 |
| Cholera | हैज़ा/कॉलरा | 13 |
| Giardia (intestinal infection) | जिआर्डिया (आंत संक्रमण) | 14 |
| Diabetes | मधुमेह | 15 |
| Hypertension | उच्च रक्तचाप | 16 |
| Asthma | दमा | 17 |
| Arthritis | गठिया | 18 |
| Pneumonia | निमोनिया | 19 |
| Cardiovascular / heart diseases | कार्डियोवैस्कुलर / हृदय रोग | 20 |
| Chronic respiratory diseases | पुरानी श्वसन रोग | 21 |
| Cancer | कैंसर | 22 |
| Urinal tract infection | मूत्र पथ संक्रमण | 23 |
| Sexually transmitted infection | यौन संचारित संक्रमण | 24 |
| HIV / AIDS | एचआईवी / एड्स | 25 |
| Others specify _________________ | अन्य स्पष्ट करें _________________ | 97 |
| Others specify _________________ | अन्य स्पष्ट करें _________________ | 98 |
| Others specify _________________ | अन्य स्पष्ट करें _________________ | 99 |

1. **What do you think are the reasons for these health problems / diseases?**

**आपके मुताबिक इन स्वास्थ्य समस्याओं / बीमारियों के कारण क्या हैं?**

**Probe – Multiple options**

unhealthy diet 1

**अस्वास्थ्यकारी आहार**

Physical inactivity 2

**शारीरिक निष्क्रियता**

Tobacco 3

**तंबाकू**

harmful use of alcohol 4

**शराब का हानिकारक उपयोग**

lack of access to fresh water 5

**ताज़े पानी की उपलब्धता नहीं होना**

Poor hygiene 6

**स्वच्छता की कमी**

poor sanitation 7

**सफाई की घटिया व्यवस्था**

high risk sexual behaviour 8

**असुरक्षित या उच्च जोखिम यौन सम्बन्ध**

lack of parental care 9

**माता-पिता की देखभाल की कमी**

others specify 99

**अन्य स्पष्ट करे**

**B. How can the community protect themselves / combat these health problems / diseases?**

**मोहल्ला(एरिया) इन स्वास्थ्य समस्याओं से / बीमारियों से / खुद को कैसे बचा सकता है?**

Healthy diet 1

**स्वस्थ आहार**

Physically active 2

**शारीरिक रूप से सक्रिय**

Avoiding Tobacco 3

**तम्बाकू का सेवन टालना**

Avoiding alcohol 4

**शराब** **का सेवन टालना**

Access to fresh water 5

**ताज़े पानी की उपलब्धता**

**Maintaining** proper hygiene 6

**उचित स्वच्छता को बनाए रखना**

**Maintaining** proper sanitation 7

**उचित सफाई को बनाए रखना**

Avoiding risky sexual behaviour 8

**जोखिम भरा या असुरक्षित यौन**  **संबंध टालना**

Proper parental care 9

**माता पिता ने उचित देखभाल करना**

others specify 99

**अन्य स्पष्ट करे**

**SCRIPTING INSTRUCTION: SHOW ONLY THOSE OPTIONS WHICH HAVE BEEN CODED IN Q15**

| **Health Problems / diseases** | स्वास्थ्य समस्याएं / बीमारियां | **Q16 A (Multiple option)** | **Q16 B (Multiple option)** |
| --- | --- | --- | --- |
| Malaria | मलेरिया |  |  |
| Dengue | डेंगू |  |  |
| Japanese Encephalitis (JE) | जापानी एन्सेफलाइटिस (जेई) |  |  |
| Chikungunya | चिकनगुनिया |  |  |
| Filaria | फाइलेरिया |  |  |
| Hookworm Infection | हुकवर्म संक्रमण |  |  |
| Influenza | इंफ्लुएंजा/ज़ुकाम |  |  |
| Jaundice | पीलिया |  |  |
| Tuberculosis | यक्ष्मा/ क्षय रोग |  |  |
| Diarrhoeal Diseases | अतिसार रोग |  |  |
| Typhoid | आंत्र ज्वर/टायफ़ायड |  |  |
| Hepatitis | हेपेटाइटिस/जिगर में सूजन |  |  |
| Cholera | हैज़ा/कॉलरा |  |  |
| Giardia (intestinal infection) | जिआर्डिया (आंत संक्रमण) |  |  |
| Diabetes | मधुमेह |  |  |
| Hypertension | उच्च रक्तचाप |  |  |
| Asthma | दमा |  |  |
| Arthritis | गठिया |  |  |
| Pneumonia | निमोनिया |  |  |
| Cardiovascular / heart diseases | कार्डियोवैस्कुलर / हृदय रोग |  |  |
| Chronic respiratory diseases | पुरानी श्वसन रोग |  |  |
| Cancer | कैंसर |  |  |
| Urinal tract infection | मूत्र पथ संक्रमण |  |  |
| Sexually transmitted infection | यौन संचारित संक्रमण |  |  |
| HIV / AIDS | एचआईवी / एड्स |  |  |
| Others specify _________________ | अन्य स्पष्ट करें _________________ |  |  |
| Others specify _________________ | अन्य स्पष्ट करें _________________ |  |  |
| Others specify _________________ | अन्य स्पष्ट करें _________________ |  |  |

| **SECTION -5: HEALTH SEEKING BEHAVIOUR** |
| --- |

1. **A) In the last one year, what are the health issues your family has suffered from?**

**पिछले एक साल में, आपके परिवार ने कौनसे स्वास्थ्य संबंधी समस्याएं का सामना किया हैं?**

**B) What was the age of the person who was affected?**

**उस व्यक्ति की आयु क्या है जो प्रभावित हुआ है?**

**C) What is the gender of the person who was affected?**

**उस व्यक्ति का क्या लिंग है जो प्रभावित हुआ है?**

**D) Which health facility did they visit to receive care?**

**देखभाल प्राप्त करने के लिए कौन सी स्वास्थ्य सुविधा को उन्होंने विजिट किया था?**

**E) On a scale of 1 to 5, where 1 is for very dissatisfied and 5 is for very satisfied, how would you rate the service received?**

**प्राप्त की हुई सेवा को आप 1 से 5 के स्केलपर किस प्रकार रेट करेंगे जहा 1 मतलब बहुत असंतुष्ट और 5 मतलब बहुत संतुष्ट**

**CAPI Instructions:**

**17A) SHOW OPTIONS OF YES = 1 AND NO = 2 USING DROPDOWN**

**17B) ALLOW THE SCRIPT TO WRITE THE AGE OF THE PERSON**

**व्यक्ति की आयु लिखने के लिए स्क्रिप्ट को अनुमति दें**

**17C) SHOW OPTIONS OF GENDER USING DROPDOWN**

**ड्रॉपडाउन का इस्तेमाल करते हुए लिंग के विकल्पों को दिखायें**

**17D) SHOW OPTIONS OF FACILITY VISITED USING DROPDOWN – Multiple option possible Option 4 is exclusive and if coded 4 skip Q17E**

**ड्रॉपडाउन का इस्तेमाल करते हुए सुविधा के विकल्पों को दिखायें – एक से अधिक विकल्प संभव**

**विकल्प 4 विशिष्ट और यदि 4 कोडेड है तो Q17E को छोडें**

**17E) ALLOW THE SCRIPT TO ENTER ANY NUMBER BETWEEN “1-5”**

| Health Issue  स्वास्थय समस्या | Yes =1 No = 2  हाँ = 1  नहीं = 2 (17a) | Age of Person Seeking Care (17c)  इलाज़ प्राप्त करने की कोशिश कर रही व्यक्ति की उम्र | Gender of Person Seeking Care  जो व्यक्ति इलाज ढूंड रही थी वह महिला थी या पुरुष (17d)  Female  महिला =1  Male पुरुष = 2 | Facility Visited (17b)  1=Private Hospital 2= Government Hospital 3= CHC  4 = Did not visit any health facility 5= Any other (please specify)  विजिट की गयी सुविधा (17b)  1=निजी हस्पताल 2= सरकारी हस्पताल 3= कम्युनिटी हेल्थ सेंटर  4 = कोई भी स्वास्थ्य सुविधा को विजिट नहीं किया 5 कोई और कृपया बताएं ) | Satisfaction with service received  प्राप्त सेवा से सुंतुष्टि (17e) | Comments  टिप्पणियाँ |
| --- | --- | --- | --- | --- | --- | --- |
| Malaria  मलेरिया | 1 |  |  | [drop down] | [drop down] |  |
|  | 2 |  |  |  |  |  |
| Dengue  डेंगू | 1 |  |  |  |  |  |
|  | 2 |  |  |  |  |  |
| Typhoid  आंत्र ज्वर/टायफ़ायड | 1 |  |  |  |  |  |
|  | 2 |  |  |  |  |  |
| Hepatitis  हेपेटाइटिस/जिगर में सूजन | 1 |  |  |  |  |  |
|  | 2 |  |  |  |  |  |
| Jaundice  पीलिया | 1 |  |  |  |  |  |
|  | 2 |  |  |  |  |  |
| Influenza/Flu  इंफ्लुएंजा/ज़ुकाम | 1 |  |  |  |  |  |
|  | 2 |  |  |  |  |  |
| Upper respiratory infection  ऊपरी श्वसन संक्रमण | 1 |  |  |  |  |  |
|  | 2 |  |  |  |  |  |
| Diarrhoea  अतिसार | 1 |  |  |  |  |  |
|  | 2 |  |  |  |  |  |
| High-blood pressure  उच्च रक्त चाप | 1 |  |  |  |  |  |
|  | 2 |  |  |  |  |  |
| Diabetes  मधुमेह | 1 |  |  |  |  |  |
|  | 2 |  |  |  |  |  |
| Tuberculosis  यक्ष्मा/ क्षय रोग | 1 |  |  |  |  |  |
|  | 2 |  |  |  |  |  |
| Asthma  दमा | 1 |  |  |  |  |  |
|  | 2 |  |  |  |  |  |
| Arthritis  गठिया | 1 |  |  |  |  |  |
|  | 2 |  |  |  |  |  |
| Pneumonia  निमोनिया | 1 |  |  |  |  |  |
|  | 2 |  |  |  |  |  |
| Musculoskeletal  हाड पिंजर रोग | 1 |  |  |  |  |  |
|  | 2 |  |  |  |  |  |
| Problems in pregnancy  गर्भावस्था में समस्याएं | 1 |  |  |  |  |  |
|  | 2 |  |  |  |  |  |
| Problems in child birth  बाल जन्म में समस्याएं | 1 |  |  |  |  |  |
|  | 2 |  |  |  |  |  |
| Cancer  कैंसर | 1 |  |  |  |  |  |
|  | 2 |  |  |  |  |  |
| HIV/AIDS  एचआईवी / एड्स | 1 |  |  |  |  |  |
|  | 2 |  |  |  |  |  |
| Others (please specify)  अन्य (कृपया स्पष्ट करें) |  |  |  |  |  |  |

1. **In general, what factors do you consider before going to a health facility?**

**आमतौर पर, स्वास्थ्य सुविधा पर जाने से पहले आप किन किन सुविधाओं का विचार करते हैं?**

**INTERVIEWER DO NOT READ REPONSE TO THE RESPONDENT:**

- **SPONTANOUS QUESTION**

**MULTIPLE CODING POSSIBLE**

| Financial resources | वित्तीय संसाधन | 1 |
| --- | --- | --- |
| Cost of services (which includes consultation and medication) | सेवाओं की लागत (जिसमें परामर्श और दवा शामिल है) | 2 |
| Cultural beliefs and practices | सांस्कृतिक मान्यता और प्रथाएँ | 3 |
| Age of the patient | मरीज की आयु | 4 |
| Perception of the cause of the illness | बीमारी के कारण की धारणा | 5 |
| Awareness on availability of medical facilities | चिकित्सा सुविधाओं की उपलब्धता पर जागरूकता | 6 |
| Sex of the patient | मरीज महिला है या पुरुष | 7 |
| Others (please specify) | अन्य, (कृपया स्पष्ट करें) |  |
| Others (please specify) | अन्य, (कृपया स्पष्ट करें) |  |
| Others (please specify) | अन्य, (कृपया स्पष्ट करें) |  |

1. **What are the reasons for why you would maybe choose to not seek health care when needed?**

**वह कौनसे कारण है जिस वजह से जरुरत पड़ने पर शायद आप स्वास्थ्य देखभाल का चयन करना पसंद नहीं करेंगे?**

**SPONTANEOUS RESPONSE**

| Distance | दूरी | 1 |
| --- | --- | --- |
| Availability/Unavailability of Services | सेवाओं की उपलब्धता / अनुपलब्धता | 2 |
| Providers behaviour | प्रदाता व्यवहार | 3 |
| Cost | लागत | 4 |
| Do not see the need | जब ऐसे लोगों को डॉक्टर को दिखाने की आवश्यकता नहीं रहती | 5 |
| Try home remedies | घरेलू उपचार का प्रयास | 6 |
| Loss of pay | वेतन का नुकसां तलने के लिए | 7 |
| Cultural Beliefs and Practices | सांस्कृतिक मान्यता और प्रथाएँ | 8 |
| Lack of confidence in care available | उपलब्ध इलाज में आत्मविश्वास की कमी |  |
| Lack of childcare in the facility | सुविधा केंद्र में बच्चों की देखभाल में कमी |  |
| Don’t know/ Not sure | पता नहीं / यकीन नहीं है | 99 |
| Others | अन्य |  |

1. **On a scale of 1-5 where 1 is not confident and 5 is very confident, how would you rate your confidence and trust in the following health providers? SINGLE CODING ONLY**

**निम्नलिखित स्वास्थ्य प्रदाताओं में आप अपने आत्मविश्वास और विश्वास को 1 से 5 के स्केलपर किस प्रकार रेट करेंगे जहा 1 मतलब आत्मविश्वास नहीं असंतुष्ट और 5 मतलब बहुत आत्मविश्वास**

| **Public health sector** |  | **1** | **2** | **3** | **4** | **5** | **98** | **99** |
| --- | --- | --- | --- | --- | --- | --- | --- | --- |
|  |  | **Not at all confident**  **बिलकुल भी भरोसा नहीं** | **Not confident**  **भरोसा नहीं** | **Somewhat confident**  **थोडा भरोसा** | **Confident**  **भरोसा** | **Very Confident**  **बहुत भरोसा** | **No idea about the facility / Don’t know**  **सुविधा के बारे में कोई आईडिया नहीं/ पता नहीं** | **Facility not avaible / NA**  **सुविधा उपलब्ध नहीं/ लागू नहीं होता** |
| Govt./municipal hospital | सरकारी / म्यूनिसिपल अस्पताल |  |  |  |  |  |  |  |
| Govt. Dispensary | सरकारी औषधालय |  |  |  |  |  |  |  |
| Vaidya/hakim/homeopath (AYUSH) | वैद्य / हाकीम / होम्योपैथ (आयुष) |  |  |  |  |  |  |  |
| Anganwadi/ICDS centre | आंगनवाड़ी / आईसीडीएस केंद्र |  |  |  |  |  |  |  |
| ASHA | आशा |  |  |  |  |  |  |  |
| Govt. Mobile clinic | सरकारी मोबाइल क्लिनिक |  |  |  |  |  |  |  |
| NGO Mobile clinic | एनजीओ मोबाइल क्लिनिक |  |  |  |  |  |  |  |
| NGO or trust hospital/clinic | एनजीओ या ट्रस्ट अस्पताल / क्लिनिक |  |  |  |  |  |  |  |
| **Private Health Sector** | निजी स्वास्थ्य क्षेत्र |  |  |  |  |  |  |  |
| Pvt. Hospital | प्रायवेट अस्पताल |  |  |  |  |  |  |  |
| Pvt. Doctor/clinic | प्रायवेट डॉक्टर / क्लिनिक |  |  |  |  |  |  |  |
| Pvt. Paramedic | प्रायवेट नर्स |  |  |  |  |  |  |  |
| Vaidya/hakim/homeopath (AYUSH) | वैद्य / हाकीम / होम्योपैथ (आयुष) |  |  |  |  |  |  |  |
| Traditional healer | पारंपरिक चिकित्सक |  |  |  |  |  |  |  |
| Pharmacy/drugstore | फार्मेसी / दवा की दुकान |  |  |  |  |  |  |  |
| Dai (TBA) | दाई (टीबीए) |  |  |  |  |  |  |  |
| Other private sector health facility | अन्य निजी क्षेत्र की स्वास्थ्य सुविधा |  |  |  |  |  |  |  |
| **Others** | अन्य |  |  |  |  |  |  |  |
| Shop | दुकान |  |  |  |  |  |  |  |
| Home treatment | गृह उपचार |  |  |  |  |  |  |  |
| **Others (please specify)** | **अन्य, (कृपया स्पष्ट करें)** |  |  |  |  |  |  |  |

1. **After medicine is prescribed to you, where do you most frequently go to get your prescription filled? SINGLE CODING ONLY**

जब दवाइयां आपके लिए निर्धारित की जाती है तब उन्हें लेने के लिए अक्सर आप कहा जाते है?

| Given by the doctor himself/herself | खुद डॉक्टर द्वारा दिया गया | **1** |
| --- | --- | --- |
| Bought from the pharmacy of the health facility visited | जिस स्वास्थ्य सुविधा में गए थे उसकी फार्मेसी से खरीदा गया | **2** |
| Bought from the pharmacy near to my household | मेरे घर के पास की फार्मेसी से खरीदा | **3** |
| Due to the unavailability of the medication locally, had to visit a pharmacy far away from the household | स्थानीय स्तर पर दवा की अनुपलब्धता के कारण, घर से दूर एक फार्मेसी जाना पड़ा | **4** |
| I do not buy medicines | मैं दवाओं को नहीं खरीदता | **5** |
| Other | अन्य |  |

1. **How much did you pay for the following the last time you went to the health facility for yourself or for someone in your family?**

**पिछली बार जब आप अपने लिए या अपने परिवार के किसी के लिए स्वास्थ्य सुविधा में गए थे, तो आपने निम्नलिखित के लिए कितना भुगतान किया था?**

**INTERVIEWER’S INSTRUCTION:**

- **RECORD THE AMOUNT IN INR**
- **WRITE IN CLEAR NUMERALS**

**SCRIPTING INSTRUCTION: CAN ACCEPT NUMERALS BETWEEN INR 1 – 10,000**

| **Health Provider consultation**  **स्वास्थ्य प्रदाता परामर्श** | **INR ________________________________**  **रूपये** |
| --- | --- |
| **Medication**  **इलाज** | **INR ________________________________**  **रूपये** |
| **Transportation**  **परिवहन** | **INR ________________________________**  **रूपये** |
| **Laboratory service**  **लैब्रटॉरी सेवा** | **INR ________________________________**  **रूपये** |
| **I Don’t Remember**  **हमें याद नहीं** |  |
| **Interviewer’s Comments (if respondent says, kids are free always or etc.)** |  |

1. **A. How did you reach the health facility last time?**

**पिछली बार आप स्वास्थ्य सुविधा तक कैसे पहुंचे?**

**INTERVIEWER’S INSTRUCTION: MULTIPLE CODING POSSIBLE**

SCRIPTING INSTRUCTION: MULTIPLE CODING POSSIBLE

| By foot | पैदल | 1 |
| --- | --- | --- |
| By motor car | मोटर कार से | 2 |
| By scooter/motorbike | स्कूटर / मोटरबाइक द्वारा | 3 |
| By metro | मेट्रो द्वारा | 4 |
| By local train | स्थानीय ट्रेन से | 5 |
| By bus | बस से | 6 |
| Others (please specify) | अन्य, (कृपया स्पष्ट करें) |  |
| Others (please specify) | अन्य, (कृपया स्पष्ट करें) |  |

1. **B. How much time did it take to reach the health facility last time?**

**पिछली बार स्वास्थ्य सुविधा तक पहुंचने में कितना समय लगा?**

**INTERVIEWER’S INSTRUCTION:**

- **RECORD THE TIME IN MINUTES**
- **WRITE IN CLEAR NUMERALS**

SCRIPTING INSTRUCTIONL TIME IN MINUTES

| **Time (in minutes)**  **समय (मिनिट्स में)** |
| --- |

| **SECTION -6: HEALTH SEEKING BEHVAIOUR OF WOMEN** |
| --- |

1. **What health needs have the women in your family experienced? (If speaking to man ask to speak to a women of reproductive age (18-49) in the family if not difficult)?**

**आपके परिवार में महिलाओं को किस स्वास्थ्य की आवश्यकता महसूस हुई? (यदि पुरुष से बात कर रहे हो तो यदि कोई परेशानी नहीं हो तो परिवार में प्रजनन आयु (18-49) की महिलाओं से बात करने के लिए कहे)?**

**SINGLE CODING FOR EACH ATTRIBUTE**

**IF THE RESPONDENT DOES NOT KNOW, CODE 99**

| **COMMON HEALTH NEEDS** | **आम स्वास्थ्य की जरूरत** | **YES**  **हाँ** | **NO**  **नहीं** | **DON’T KNOW/CAN’T SAY**  **मालूम नहीं / कह नहीं सकते** |
| --- | --- | --- | --- | --- |
| Urinary tract infections | मूत्र मार्ग में संक्रमण | 1 | 2 | 99 |
| Aneamia | खून की कमी | 1 | 2 | 99 |
| Cancer | कैंसर | 1 | 2 | 99 |
| HIV/AIDS | एचआईवी / एड्स | 1 | 2 | 99 |
| Cardiac problems | ह्रदय/ दिल से जुड़ी समस्याएं | 1 | 2 | 99 |
| Hypertension | उच्च रक्तचाप | 1 | 2 | 99 |
| Diabetes | मधुमेह | 1 | 2 | 99 |
| Menstrual Health | मासिक धर्म स्वास्थ्य | 1 | 2 | 99 |
| Uterus infection | गर्भाशय जंतु संक्रमण | 1 | 2 | 99 |
| Iodine deficiency | आयोडीन की कमी | 1 | 2 | 99 |
| Use of contraceptives | गर्भ निरोधकों का उपयोग | 1 | 2 | 99 |
| Vaginal infection | योनि में जंतु संक्रमण | 1 | 2 | 99 |
| Others (please specify) | अन्य, (कृपया स्पष्ट करें) |  |  |  |
| Others (please specify) | अन्य, (कृपया स्पष्ट करें) |  |  |  |

1. **Irrespective of the outcome of the pregnancy, can you please tell me how many times did you become pregnant in your life time?**

**इस बात की परवाह किया बिना कि गर्भधारण का परिणाम क्या हुआ, कृपया मुझे बताएं कुलमिलाकर कितनीबार आप अपने पूरे जीवन में गर्भावस्था में थी?**

**INTERVIEWER’S INSTRUCTION:**

**OPEN ENDED, WRITE THE ANSEWR IN THE SPACE PROVIDED**

**CRIPTING INSTRUCTION: OPEN ENDED**

**ALLOW NUMERALS FROM 0 -15**

| **Total number of pregnancies __________________________**  **गर्भावस्था की कुल संख्या _____________________** |
| --- |

**SCRIPTING INSTRUCTION: SKIP TO NEXT SECTION IF Q25 IS “0”**

1. **How many live births did you have?**

**आपने कितने जीवित बच्चो को जन्म दिया है?**

**INTERVIEWER’S INSTRUCTION:**

**OPEN ENDED**

**WRITE THE ANSEWR IN THE SPACE PROVIDED**

**SCRIPTING INSTRUCTION: OPEN ENDED**

**ALLOW NUMERALS FROM 0 -15**

| **Number of live births given ___________________________________________-**  **कुल जीवित बच्चो का जन्म** |
| --- |

1. **How many times did you go for ANC checkups the last time you were pregnant?**

**पिछली बार आप गर्भवती होने पर आप एएनसी चेकअप के लिए कितनी बार गयी थी?**

**INTERVIEWER’S INSTRUCTION: SINGLE CODING ONLY, Only ask if woman has kids**

SCRIPTING INSTRUCTION: SINGLE CODING ONLY

| 0 | 1 |
| --- | --- |
| 1 | 2 |
| 2 | 3 |
| 3 | 4 |
| 4 | 5 |
| More than 4 times | 6 |

1. **Where did you go for the most of your ANC check-ups?**

**आपके अधिकतर एएनसी चेकअप के लिए आप कहा गयें थे?**

**INTERVIEWER’S INSTRUCTION: SINGLE CODING ONLY**

SCRIPTING INSTRUCTION: SINGLE CODING ONLY

| Government Health Facility  **सरकारी स्वास्थ्य सुविधा** | 1 |
| --- | --- |
| Private Health Facility  **निजी स्वास्थ्य सुविधा** | 2 |
| Any other (please specify)  कोई अन्य, (कृपया स्पष्ट करें) |  |

1. **Did anyone tell you to visit a health facility in case you experienced pregnancy related complications? SINGLE CODING ONLY**

**क्या आपको गर्भावस्था से संबंधित जटिलताओं का सामना करने के मामले में किसीने स्वास्थ्य सुविधा पर जाने के लिए कहा गया है?**

| Yes  हाँ | 1 |
| --- | --- |
| No  नहीं | 2 |

1. **A) Did you face any health problems during your last pregnancy?**

**क्या आपको आपकी पिछली गर्भावस्था के दौरान किसी भी स्वास्थ्य समस्याओं का सामना करना पड़ा?**

| Yes  हाँ | 1 |
| --- | --- |
| No  नहीं | 2 |

**CAPI INSTRUCTIONS: IF CODED YES, THEN ASK 29b**

**B) If yes, which health issues did you face during pregnancy?**

**यदि हाँ तो गर्भावस्था के दौरान आपको कौनसे स्वास्थ्य समस्याओं का सामना करना पड़ा?**

| **HEALTH ISSUES FACED DURING PREGNANCY** | **स्वास्थ्य समस्याए गर्भावस्था के दौरान** | **YES**  **हाँ** | **NO**  **नहीं** |
| --- | --- | --- | --- |
| High blood pressure | उच्च रक्त चाप | 1 | 2 |
| Gestational diabetes | गर्भावधि मधुमेह | 1 | 2 |
| Anaemia | खून की कमी | 1 | 2 |
| No increase in body weight of the mother | मां के शरीर के वजन में कोई वृद्धि नहीं | 1 | 2 |
| Vaginal Bleeding | योनि रक्तस्राव | 1 | 2 |
| Convulsions | ऐंठन | 1 | 2 |
| Prolonged Labour | लंबा प्रसव-काल | 1 | 2 |
| Any other (please specify) | कोई अन्य (कृपया स्पष्ट करें) |  | |
| Any other (please specify) | कोई अन्य (कृपया स्पष्ट करें) |  | |

1. **Did you experience any health problems within 6 weeks after your last delivery?**

**क्या आपने आपकी पिछली डिलीवरी के 6 सप्ताह के भीतर किसी भी स्वास्थ्य समस्याएं का अनुभव किया था?**

| Yes  हाँ | 1 |
| --- | --- |
| No  नहीं | 2 |

**CAPI INSTRUCTIONS: IF CODED YES, THEN ASK 30B AND 31, ELSE SKIP TO Q32**

1. **B) If yes, which health issues did you face during pregnancy?**

**यदि हां तो गर्भावस्था के दौरान आपको किस स्वास्थ्य समस्या का सामना करना पड़ा?**

**SINGLE CODING ONLY FOR EACH HEALTH PROBLEM**

| **HEALTH PROBLEMS** | **स्वास्थ्य समस्याएं** | **YES**  **हाँ** | **NO**  **नहीं** |
| --- | --- | --- | --- |
| High fever | तेज बुखार | 1 | 2 |
| Lower abdominal pain | पेट के नीचले भाग में दर्द | 1 | 2 |
| Foul smelling vaginal discharge | योनि निर्वहन से दुर्गंध | 1 | 2 |
| Excessive bleeding | अधिकतम खून बहना | 1 | 2 |
| Convulsions | ऐंठन | 1 | 2 |
| Severe headache | भयानक सरदर्द | 1 | 2 |
| Urinary Incontinence | मूत्र असंयम | 1 | 2 |
| Uterus infection | गर्भाशय संक्रमण | 1 | 2 |
| Hemorrhoids | बवासीर | 1 | 2 |
| Others (specify)  अन्य (बताएं) |  | 1 | 2 |
| Others (specify)  अन्य (बताएं) |  | 1 | 2 |

1. **Did you visit a health facility for any of these complications?**

**क्या आप इनमें से किसी भी समस्याओं के लिए स्वास्थ्य सुविधा पर गए थे?**

**SINGLE CODING ONLY**

| Yes  हाँ | 1 |
| --- | --- |
| No  नहीं | 2 |

1. **Which of the following family planning methods do you use?**

आप निम्नलिखित में से किन परिवार नियोजन विधियों का उपयोग करते हैं?

- **MULTIPLE OPTION POSSIBLE**

**एक से अधक विकल्प संभव**

- EXCLUSIVE CODING OF NONE OF THE ABOVE

| Family Planning Methods | परिवार नियोजन के तरीके | Codes |
| --- | --- | --- |
| Condoms | कंडोम | 1 |
| Copper-T | कॉपर-टी | 2 |
| Pills | गोलियां | 3 |
| IUD | आईयूडी | 4 |
| Male Sterilization | पुरुष नसबंदी | 6 |
| Female Sterilization | महिला नसबंदी | 7 |
| Do not follow / use any family planning method | कोई भी परिवार नियोजन के तरीके का पालन नहीं किया/ इस्तेमाल नहीं किया |  |
| Any other (please specify) | कोई अन्य (कृपया स्पष्ट करें) |  |

**IF CODED “NO” IN Q32, THEN ASK Q33**

**33 b. Have you ever got pregnant while using a specific family planning method, basically the failure of family planning method that you have used?**

एक विशिष्ट परिवार नियोजन का तरीका इस्तेमाल करते हुए क्या आप कभी गर्भवती हुई हैं, वस्तुतः उस परिवार नियौजन के तरीके के फेल होने के कारण जिसका आपने इस्तेमाल किया था?

**Scripter instruction: show only the options that got coded ‘yes’ in Q33**

**स्क्रिप्टर को निर्देश: केवल उन विकल्पों को दिखायें जो Q33 में ‘हाँ’ कोडेड है**

| Family Planning Methods  **परिवार नियोजन के तरीके** | Yes  **हाँ** | No  **नहीं** |
| --- | --- | --- |
| Condoms  **कंडोम** | 1 | 2 |
| Copper-T  **कॉपर टी** | 1 | 2 |
| Pills  **दवाएँ** | 1 | 2 |
| IUD  **आईयुडी** | 1 | 2 |
| Male Sterilization  **पुरुष नसबंदी** | 1 | 2 |
| Female Sterilization  **स्त्री नसबंदी** | 1 | 2 |
| Any other (please specify)  **कोई अन्य (कृपया स्पष्ट करें)** | 1 | 2 |

1. **What obstacles do you face in using family planning methods?**

परिवार नियोजन विधियों का उपयोग करने में आपको किस बाधा का सामना करना पड़ता है?

**MULTIPLE CODING POSSIBLE**

| Husband refuses to use a condom | पति कंडोम का उपयोग करने से इंकार कर देता है | 1 |
| --- | --- | --- |
| Mother in law does not allow to use any family planning methods | सास किसी परिवार नियोजन विधियों का उपयोग करने की अनुमति नहीं देती है | 2 |
| Afraid to use them as they may cause further complications | उनका उपयोग करने से डरते हैं क्योंकि वह आगे समस्याओं का कारण बन सकते हैं | 3 |
| Cannot afford family planning methods | परिवार नियोजन विधियों का खर्च नहीं उठा सकते है | 4 |
| Used a family planning method in past, but it was a failure as I/wife became pregnant | अतीत में एक पारिवार नियोजन विधि का उपयोग किया था, लेकिन यह विफलता थी क्योंकि मैं / पत्नी गर्भवती हो गई थी | 5 |
| Do not have access to family planning methods | परिवार नियोजन विधियों की उपलब्धता नहीं है | 6 |
| Not aware about family planning methods | परिवार नियोजन विधियों के बारे में पता नहीं है | 7 |
| Otherrs (Specify) |  |  |

| **SECTION -7: HEALTH SEEKING BEHVAIOUR FOR CHILDREN** |
| --- |

**SCRIPTING INSTRUCTION: FILTER FROM Q7 (children below 5 years of age)**

1. **A. Did any child in your household suffered from diarrhoea in last 2 weeks in your house? SINGLE CODING ONLY**

**क्या आपके घर में पिछले 2 हफ्तों में कोईभी भी बच्चा दस्त से पीड़ित था?**

| Yes  हाँ | 1 |
| --- | --- |
| No  नहीं | 2 |

**CAPI Instruction: IF CODED YES IN 33A, ASK 33B**

**B. If Yes, what was the age of the child?**

**यदि हां तो उस बच्चे की उम्र क्या थी?**

| **If Yes,**  यदि हाँ | **Age**  उम्र |
| --- | --- |
| Child 1  बच्चा 1 |  |
| Child 2  बच्चा 2 |  |
| Child 3  बच्चा 3 |  |
| Child 4  बच्चा 4 |  |
| Child 5  बच्चा 5 |  |

1. **A Do you take your children for well-child visits (prompt what well-child visits are)?**

**SINGLE CODING ONLY**

**क्या आप आपके बच्चे को अच्छे स्वास्थ्य के लिए कही ले गए थे? (पूछे कौनसे अच्छे स्वास्थ्य के लिए)**

| Yes  हाँ | 1 |
| --- | --- |
| No  नहीं | 2 |

**CAPI Instruction: IF CODED YES IN 35A, ASK 35B**

**B. If yes, to which health facility do you take your child to?**

**यदि हाँ तो आप अपने बच्चे को कौनसे स्वास्थ्य सुविधा के लिए गए थे?**

| **If Yes,**  **यदि हाँ,** | **Health Facility Visited**  कौनसे **स्वास्थ्य सुविधा** |
| --- | --- |
| Visit 1  विजिट 1 |  |
| Visit 2  विजिट 2 |  |
| Visit 3  विजिट 3 |  |

1. **What are the health problems seen among children in your household?**

**आपके घर में बच्चों के बीच कौनसी स्वास्थ्य समस्याएं देखी गयी हैं?**

**SPONTANEOUS RESPONSE, MULTIPLE RESPONSE**

| DISEASES | बीमारियाँ | SPONT | PROBE |
| --- | --- | --- | --- |
| Asthma | दमा | 1 | 1 |
| Pneumonia | निमोनिया | 2 | 2 |
| Respiratory Infection | श्वसन संक्रमण | 3 | 3 |
| Diphtheria | डिप्थीरिया | 4 | 4 |
| Dengue | डेंगू | 5 | 5 |
| Malaria | मलेरिया | 6 | 6 |
| Measles | खसरा | 7 | 7 |
| Aneamia | खून की कमी | 8 | 8 |
| Epilepsy | मिरगी | 9 | 9 |
| Tuberculosis (TB) | क्षय रोग (टीबी) | 10 | 10 |
| Underweight/malnutrition | कम वजन / कुपोषण | 10 | 10 |
| No health issues | कोई स्वास्थ्य समस्या नहीं | 11 | 11 |
| Don’t know / can’t say | पता नहीं/ बता नहीं सकते | 98 | 98 |
| Any other (please specify)_______________ | कोई अन्य (कृपया स्पष्ट करें) _______________ | 12 | 12 |
| Any other (please specify)_______________ | कोई अन्य (कृपया स्पष्ट करें) _______________ | 13 | 13 |
| Any other (please specify)_______________ | कोई अन्य (कृपया स्पष्ट करें) _______________ | 14 | 14 |
| Any other (please specify)_______________ | कोई अन्य (कृपया स्पष्ट करें) _______________ | 15 | 15 |

1. **What preventive measures do you take to protect the child from these health problems? SPONTANEOUS RESPONSE**

**बच्चे को इन स्वास्थ्य समस्याओं से बचाने के लिए आप क्या निवारक उपाय करते हैं?**

| Purification of drinking water | पीने के पानी का शुद्धिकरण | 1 |
| --- | --- | --- |
| Keeping the surroundings clean | आसपास के वातावरण को साफ रखना | 2 |
| Properly dressing the child during monsoon season (i.e. the child is fully covered with no body parts exposed) | बारिश के मौसम के दौरान बच्चे को उचित रूप से तैयार करना (यानी बच्चे को पुरे शरीर के अंगों को ढकना कोईभी अंग खुला नहीं छोड़ना) | 3 |
| Proper nutrition of the child | बच्चे का उचित पोषण | 4 |
| Handwashing | हाथ धोना | 5 |
| Discouraging Open Defecation | खुले में सौच के लिए मना करना | 6 |
| Any other (please specify) | पीने के पानी का शुद्धिकरण |  |
| Any other (please specify) | कोई अन्य (कृपया स्पष्ट करें) _______________ |  |
| Any other (please specify) | कोई अन्य (कृपया स्पष्ट करें) _______________ |  |

| **SECTION -8: HEALTH SEEKING BEHVAIOUR FOR ELDERLY** |
| --- |

**SCRIPTING INSTRUCTION: FILTER FROM Q7 (60+ years of age), IF NO ELDERLY IN Q7, SKIP SECTION**

1. **What are the health problems that the elderly are suffering from, in your household? MULTIPLE CODING POSSIBLE**

**आपके घर के बुजर्ग किन स्वास्थ्य समस्याओं से पीड़ित है?**

| Blood Pressure |  | रक्त चाप | 1 |
| --- | --- | --- | --- |
| Diabetes |  | मधुमेह | 2 |
| Heart Disease |  | दिल की बीमारी | 3 |
| Arthritis |  | गठिया | 4 |
| Cancer |  | कैंसर | 5 |
| Respiratory Disease |  | श्वसन संबंधी रोग | 6 |
| Alzheimer’s Disease |  | अल्जाइमर रोग | 7 |
| Osteoporosis |  | ऑस्टियोपोरोसिस | 8 |
| Depression |  | डिप्रेशन | 9 |
| Stroke |  | आघात/स्ट्रोक | 10 |
| No health issues |  | कोई भी स्वास्थ्य की समस्या नहीं | 98 |
| Any other (please specify) |  | कोई अन्य (कृपया स्पष्ट करें) _______________ |  |
| Any other (please specify) |  | कोई अन्य (कृपया स्पष्ट करें) _______________ |  |
| Any other (please specify) |  | कोई अन्य (कृपया स्पष्ट करें) _______________ |  |
| Any other (please specify) |  | कोई अन्य (कृपया स्पष्ट करें) _______________ |  |
| Any other (please specify) |  | कोई अन्य (कृपया स्पष्ट करें) _______________ |  |
| Any other (please specify) |  | कोई अन्य (कृपया स्पष्ट करें) _______________ |  |

1. **Did they face any emergencies in the last 6 months? SINGLE CODING ONLY**

**क्या उन्हें पिछले 6 महीनों में किसी भी आपात स्थिति का सामना करना पड़ा?**

| Yes  हाँ | 1 |
| --- | --- |
| No  नहीं | 2 |

1. **What type of emergency occurred? SPONTANEOUS RESPONSE**

**कौनसे प्रकार की आपात स्थिति हो गयी थी?**

| Breathing difficulty | सांस लेने में कठिनाई | 1 |
| --- | --- | --- |
| Seizure | मिर्गी | 2 |
| Chest Pain | छाती में दर्द | 3 |
| Abdominal Pain | पेट में दर्द | 4 |
| Stroke | आघात | ~~5~~ |
| Fall | गिरना | 6 |
| Altered Sensorium | भ्रमित होना/ आजुबाजु की परिस्थिति का ज्ञान खो देना | 7 |
| Any other (please specify) | कोई अन्य (कृपया स्पष्ट करें) _______________ |  |

| **SECTION -9: AWARENESS ABOUT MENTAL HEALTH AND MENTAL ILLNESS** |
| --- |

1. **What are the most common causes of stress in the community, for both men and women in your community? SPONTANEOUS RESPONSE**

**मोहल्ला(एरिया) में तनाव के सबसे आम कारण क्या हैं, आपके मोहल्ला(एरिया) के पुरुषों और महिलाओं दोनों में?**

| Family Issues | पारिवारिक समस्याएं | 1 |
| --- | --- | --- |
| Unemployment | बेरोजगारी | 2 |
| Repeated failures | बार-बार विफलताएं | 3 |
| Stress due to financial problems | वित्तीय समस्याओं के कारण तनाव | 4 |
| Stress related to work | काम से संबंधित तनाव | 5 |
| Not able to make a place in the preferred peer group | पसंदीदा सहकर्मी समूह में जगह बनाने में सक्षम नहीं है | 6 |
| Prolonged illness of an elderly | बुजुर्गों की पुरानी/लंबी बीमारी | 7 |
| Prolonged illness of spouse | पति / पत्नी की लंबी बीमारी | 8 |
| Prolonged illness of child | बच्चे की लंबी बीमारी | 9 |
| Prolonged illness of self | स्वयं की लंबी बीमारी | 10 |
| Managing multiple responsibilities | कई जिम्मेदारियों का प्रबंधन | 11 |
| Don’t know / can’t say | पता नहीं/ कह नहीं सकते | 98 |
| Any other (please specify) | कोई अन्य (कृपया स्पष्ट करें) _______________ |  |
| Any other (please specify) | कोई अन्य (कृपया स्पष्ट करें) _______________ |  |
| Any other (please specify) | कोई अन्य (कृपया स्पष्ट करें) _______________ |  |
| Any other (please specify) | कोई अन्य (कृपया स्पष्ट करें) _______________ |  |

1. **What are the coping mechanisms that people in the community have to cope with stress?**

**मुकाबला करने वाले वह कौनसे तंत्र क्या हैं जिनका इस्तमाल मोहल्ला(एरिया) में लोगों ने तनाव का सामना करने के लिए करना चाहिएं?**

- **MULTIPLE CODING POSSIBLE**
- IF CODED “DON’T KNOW/CAN’T SAY THEN NO OTHER OPTION CAN BE CODED

| Do a positive activity | सकारात्मक गतिविधि करे | 1 |
| --- | --- | --- |
| Positive self-talk | सकारात्मक आत्म-चर्चा | 2 |
| Socialize with friends and relatives | दोस्तों और रिश्तेदारों के साथ मिलना जुलना | 3 |
| Visit a mental health provider | एक मानसिक स्वास्थ्य प्रदाता से मिले | 4 |
| Religious/Spiritual Activity | धार्मिक / आध्यात्मिक गतिविधि | 5 |
| Don’t Know/Can’t Say | पता नहीं / कह नहीं सकते | 6 |
| Any other (please specify) | कोई अन्य (कृपया स्पष्ट करें) _______________ |  |
| Any other (please specify) | कोई अन्य (कृपया स्पष्ट करें) _______________ |  |

1. **Are you aware of anyone in the community who is experiencing one of the following? SINGLE CODING ONLY**

**आपके मोहल्ला(एरिया) में आप किसी को जानते हैं जो निम्न में से किसी एक से पीड़ित हो?**

|  |  | YES  हाँ | NO  नहीं | Don’t know / can’t say  पता नहीं / कह नहीं सकते |
| --- | --- | --- | --- | --- |
| Anxiety | चिंता | 1 | 2 | 98 |
| Stress | तनाव | 1 | 2 | 98 |
| Depression | डिप्रेशन | 1 | 2 | 98 |
| More severe mental illness | अधिक गंभीर मानसिक बीमारी | 1 | 2 | 98 |

1. **Have you heard of any suicides, or attempted suicides, in this community? SINGLE CODING**

**क्या आपने इस मोहल्ला(एरिया) में किसी भी आत्महत्या, या आत्महत्या की कोशिश के बारेमे सुना है?**

| Yes हाँ | 1 |
| --- | --- |
| No नहीं | 2 |

1. **Are you aware of any mental health services being provided in your community or a nearby community? SINGLE CODING ONLY**

**क्या आप अपने मोहल्ला(एरिया) या पास के मोहल्ला(एरिया) में उपलब्ध किसी भी मानसिक स्वास्थ्य सेवाओं से अवगत हैं**?

| Yes हाँ | 1 |
| --- | --- |
| No नहीं | 2 |
| Don’t Know/Not Sure  पता नहीं / यकीन से नहीं कह सकते | 3 |

1. **How do you or people in your community extend help to people who are suffering from mental illness?**

**आप या आपके मोहल्ला(एरिया) के लोग मानसिक बीमारी से पीड़ित लोगों को कैसे मदद करते हैं?**

**MULTIPLE CODING POSSIBLE**

**IF CODED “DON’T KNOW/CAN’T SAY THEN NO OTHER OPTION CAN BE CODED**

| Listen to their problems and give advice | उनकी समस्याओं को सुनते है और सलाह देते है | 1 |
| --- | --- | --- |
| Keep in touch with them frequently | अक्सर उनके साथ संपर्क में रहते है | 2 |
| Suggest visiting a health provider | एक स्वास्थ्य प्रदाता का दौरा करने का सुझाव देते है | 3 |
| Bringing them into companionship of good friends | उन्हें अच्छे दोस्तों के संपर्क में लाते है | 4 |
| Don’t help at all | बिल्कुल मदद नहीं करते | 99 |
| Any other (please specify) | कोई अन्य (कृपया स्पष्ट करें) _______________ |  |
| Any other (please specify) | कोई अन्य (कृपया स्पष्ट करें) _______________ |  |
| Don’t know / can’t say | पता नहीं / कह नहीं सकते | 98 |

| **SECTION -10: VIOLENCE IN THE COMMUNITY** |
| --- |

1. **What are the common forms of violence in the community?**

**मोहल्ला(एरिया) में हिंसा के आम रूप क्या हैं?**

**MULTIPLE CODING POSSIBLE**

**IF CODED “DON’T KNOW/CAN’T SAY THEN NO OTHER OPTION CAN BE CODED**

| Physical Violence | शारीरिक हिंसा | 1 |
| --- | --- | --- |
| Psychological violence | मानसिक हिंसा | 2 |
| Sexual Violence | यौन हिंसा | 3 |
| No violence in the community | समाज में कोई हिस्सा नहीं | 4 |
| Don’t know/Can’t Say | पता नहीं / कह नहीं सकते | 98 |

**Scripting instruction: Show only the codes choosen in Q48 for thte remaining question of this section. If coded 4 or 98 skip this section**

**स्क्रिप्टर को निर्देश: इस सेक्शन के बचे हुए प्रश्न के लिए केवल Q48 में चुने गए कोड्स को दिखायें| इस सेक्शन को छोडें यदि 4 या 98 कोडेड है|**

1. **What are the common examples of the following violence in the community?**

**मोहल्ला(एरिया) में निम्नलिखित हिंसा के आम उदाहरण क्या हैं?**

- **OPEN ENDED RESPONSE**
- **IF THE RESPONDENT DOES NOT KNOW, THEN CODE 99**

| **Forms of Violence** | **हिंसा के रूप** | Common Examples  आम उदाहारण | Don’t Know/Can’t Say  पता नहीं / कह नहीं सकते |
| --- | --- | --- | --- |
| Physical Violence | शारीरिक हिंसा |  | 99 |
| Psychological violence | मानसिक हिंसा |  | 99 |
| Sexual Violence | यौन हिंसा |  | 99 |
| Don’t know/Can’t Say | पता नहीं / कह नहीं सकते |  | 99 |

1. **Which gender suffers from the following forms of violence and why?**

**महिला या पुरुष कौन हिंसा के निम्नलिखित रूपों से पीड़ित हैं और क्यों?**

- **CAN ADD AGE GROUP UPTO 80 YEARS OF AGE**
- **REASONS SHOULD BE OPEN ENDED**

| **Forms of Violence** | **हिंसा के रूप** | **Gender who suffers from violence the most Males = 1 Females = 2**  कौन सबसे अधिक हिंसा से पीड़ित है  पुरुष = 1  महिला = 2 | **Reasons**  कारण |
| --- | --- | --- | --- |
| Physical Violence | शारीरिक हिंसा |  |  |
| Psychological violence | मानसिक हिंसा |  |  |
| Sexual Violence | यौन हिंसा |  |  |
| Don’t know/Can’t Say | पता नहीं / कह नहीं सकते |  |  |

1. **On a scale of 1-5, where “1” stands for “not at all frequent” and “5” stands for “very frequent”, what is the frequency of different forms of violence?**

**1 – 5 के स्केल पर हिंसा के विभिन्न रूपों की आवृत्ति क्या है? जहा “1” का मतलब है “बिलकुल भी नहीं” और ‘5’ का मतलब “बहुत अक्सर”**

| **Forms of Violence** | **हिंसा के रूप** | **Frequency of Violence**  हिंसा की **आवृत्ति** |
| --- | --- | --- |
| Physical Violence | शारीरिक हिंसा |  |
| Psychological violence | मानसिक हिंसा |  |
| Sexual Violence | यौन हिंसा |  |

| **SECTION -11: SUBSTANCE ABUSE AND ADDICTION** |
| --- |

1. **How common are these types of addiction in your community? SINGLE CODING ONLY FOR EACH ATTRIBUTE**

**आपके मोहल्ला(एरिया) में इन प्रकार की लत कितनी आम है?**

|  | Rarely (1)  बहुत कम | Sometimes (2)  कभीकभार | Frequently (3)  अक्सर | Addiction does not excist in the community  समाज में लत नहीं मौजूद नहीं है |
| --- | --- | --- | --- | --- |
| Tobacco  सिगरेट, बीड़ी | 1 | 2 | 3 | 98 |
| Alcohol  शराब | 1 | 2 | 3 | 98 |
| Drugs  ड्रग्स | 1 | 2 | 3 | 98 |
| Gutka  गुटखा | 1 | 2 | 3 | 98 |
| Any other (please specify)______________  कोई अन्य (कृपया स्पष्ट करें) _______________ | 1 | 2 | 3 | 98 |

1. **What are the symptoms of substance abuse? SPONTANEOUS RESPONSE**

**पदार्थों के दुरुपयोग के लक्षण क्या हैं?**

| Stress | तनाव | 1 |
| --- | --- | --- |
| Depression/Anxiety | अवसाद / चिंता | 2 |
| Decreased appetite | कम हुई भूख | 3 |
| Weight loss | वजन घटना | 4 |
| Stroke | आघात | 5 |
| Paranoia and hallucination | मानसिक उन्माद और दृष्टिभ्रम | 6 |
| Memory loss | स्मरण शक्ति की क्षति | 7 |
| Heart Failure | ह्रदय की धडकन रुक जाना | 8 |
| Sleeplessness | उन्निद्रता | 9 |
| Aggressive Behavior | आक्रामक व्यवहार | 10 |
| Coughing | खाँसी | 11 |
| Don’t know / can’t say | पता नहीं / कह नहीं सकते | 98 |
| Any other (please specify) | कोई अन्य (कृपया स्पष्ट करें) _______________ |  |
| Any other (please specify) | कोई अन्य (कृपया स्पष्ट करें) _______________ |  |
| Any other (please specify) | कोई अन्य (कृपया स्पष्ट करें) _______________ |  |
| Any other (please specify) | कोई अन्य (कृपया स्पष्ट करें) _______________ |  |

1. **Do you know of somebody who is not able to give up their addiction? SINGLE CODING ONLY**

**क्या आप किसी ऐसे व्यक्ति के बारे में जानते हैं जो अपनी लत छोड़ने में सक्षम नहीं है?**

| Yes हाँ | 1 |
| --- | --- |
| No नहीं | 2 |

1. **Are you aware of any treatment program that is being/was being provided in your community to combat addiction? SINGLE CODING ONLY**

**क्या आप किसी भी उपचार कार्यक्रम से अवगत हैं जो आपके मोहल्ला(एरिया) में व्यसन/लत छुड़ाने के लिए उपलब्ध कराया जा रहा है/था?**

| Yes हाँ | 1 |
| --- | --- |
| No नहीं | 2 |

| **SECTION -12: AVERAGE ANNUAL EXPENSE ON HEALTH** |
| --- |

1. **Are you aware of any government health insurance scheme? SINGLE CODING ONLY**

**क्या आप किसी भी सरकारी स्वास्थ्य बीमा योजना से अवगत हैं?**

| Yes हाँ | 1 |
| --- | --- |
| No नहीं | 2 |
| Don’t Know/Can’t Say  पता नहीं/कह नहीं सकते | 3 |

1. **How many household members are covered in any health scheme or health insurance? SINGLE CODING ONLY**

**आपके घर के कितने सदस्यों को कोई भी स्वास्थ्य योजना या स्वास्थ्य बीमा में शामिल किया गया है?**

| Type Response  जवाब टाइप करे | ____________ |
| --- | --- |
| Don’t Know/Can’t Say  पता नहीं/कह नहीं सकते |  |

1. **Did you face any problem in availing the insurance scheme?**

**SINGLE CODING ONLY**

**क्या आपको बीमा योजना का लाभ उठाने में कोई समस्या आती है?**

| Yes हाँ | 1 |
| --- | --- |
| No नहीं | 2 |
| Don’t Know/Can’t Say  पता नहीं/कह नहीं सकते | 3 |

1. **How much did you pay / spend for you and your HH members last year (in INR) on medicine AND medical consultations? ALLOW NUMERALS BETWEEN 1-10,00,000**

**पिछले साल आपने दवाइयां और स्वास्थ्य परामर्श पर कितना भुगतान किया था (रूपये में)/आपके लिए और आपके घर के सदस्यों के लिए ?**

| **Medicines Average expenditure (in INR) _____________________________________** | **दवाएं पर औसत खर्च (रूपये में)_____________________________________** |
| --- | --- |
| **I don’t know** | **मुझे नहीं पता** |
| **Consultations Average expenditure (in INR) _____________________________________** | **परामर्श पर औसत खर्च (रूपये में)_____________________________________** |
| **I don’t know** | **मुझे नहीं पता** |

| **SECTION -12: AWARENESS OF AC MOBILE HEALTH CENTRES OR ANY MHCS** |
| --- |

1. **Are you aware of Mobile Health Center services? SINGLE CODING ONLY**

**क्या आप मोबाइल हेल्थ सेंटर सेवाओं से अवगत हैं?**

| Yes  हाँ | 1 |
| --- | --- |
| No  नहीं | 2 |

**INSTRUCTION: IF CODED “2” END THE INTERVIEW AND THANK THE RESPONDENT**

**सुचना : यदि “2” कोड़े किया हो तो रिस्पोंड़ेन्ट का धन्यवाद कह कर इन्टरव्यू समाप्त करे**

1. **Can you tell me the names of the ones you are aware of?**

**क्या उन में से किसी एक का नाम आप हमें बता सकते है जिससे आप अवगत हो?**

| MHC 1  एमएचसी 1 | __________________ |
| --- | --- |
| MHC 2  एमएचसी 2 | ______________________________ |
| MHC 3  एमएचसी 3 |  |
| Don’t Know  मालूम नहीं | 3 |

1. **From where did you hear about Mobile Health Center? SINGLE CODING ONLY**

**मोबाइल हेल्थ सेंटर के बारे में आपने कहां से सुना?**

| Through community health workers | सामुदायिक स्वास्थ्य कर्मियों के माध्यम से | 1 |
| --- | --- | --- |
| Through friends | दोस्तों के माध्यम से | 2 |
| Through government doctors and nurses | सरकारी डॉक्टरों और नर्सों के माध्यम से | 3 |
| Through community head | सामुदायिक सिर के माध्यम से | 4 |
| Through family and relatives | परिवार और रिश्तेदारों के माध्यम से | 5 |
| Any other (please specify) | कोई अन्य (कृपया स्पष्ट करें) |  |

1. **Have you ever used/ accessed MHC services? SINGLE CODING ONLY**

**क्या आपने कभीभी एमएचसी सेवाओं का उपयोग / प्रयोग किया है?**

| Yes  हाँ | 1 |
| --- | --- |
| No  नहीं | 2 |

**INSTRUCTION: IF CODED “2” END THE INTERVIEW AND THANK THE RESPONDENT**

**सुचना : यदि “2” कोड़े किया हो तो रिस्पोंड़ेन्ट का धन्यवाद कह कर इन्टरव्यू समाप्त करे**

1. **For what health issues did you visit the MHC? SINGLE CODING FOR EACH HEALTH ISSUE**

**आप किस स्वास्थ्य समस्याओं को लेकर एमएचसी गए थे?**

| Diseases | बीमारियाँ | Yes  हाँ | No  नहीं | If yes, How Long Ago  यदि हाँ तो कितने समय से  (Mention in days)  (दिनों में बताएं)  [Drop Down] |
| --- | --- | --- | --- | --- |
| Malaria | मलेरिया | 1 | 2 |  |
| Dengue | डेंगू | 1 | 2 |  |
| Typhoid | आंत्र ज्वर | 1 | 2 |  |
| Hepatitis | हेपेटाइटिस | 1 | 2 |  |
| Jaundice | पीलिया | 1 | 2 |  |
| Influenza/Flu | इन्फ्लुएंजा / फ्लू | 1 | 2 |  |
| Upper respiratory infection | ऊपरी श्वसन संक्रमण | 1 | 2 |  |
| Diarrhoea | दस्त | 1 | 2 |  |
| High-blood pressure | उच्च रक्त चाप | 1 | 2 |  |
| Diabetes | मधुमेह | 1 | 2 |  |
| Tuberculosis | यक्ष्मा/क्षय रोग | 1 | 2 |  |
| Asthma | दमा | 1 | 2 |  |
| Arthritis | गठिया | 1 | 2 |  |
| Pneumonia | निमोनिया | 1 | 2 |  |
| Musculoskeletal | हाड पिंजर रोग | 1 | 2 |  |
| Problems in pregnancy | गर्भावस्था में समस्याएं | 1 | 2 |  |
| Problems in child birth | बाल जन्म में समस्याएं | 1 | 2 |  |
| Cancer | कैंसर | 1 | 2 |  |
| HIV/AIDS | एचआईवी / एड्स | 1 | 2 |  |
| Others (please specify) | अन्य, कृपया स्पष्ट करें) |  |  |  |
| Others (please specify) | अन्य, कृपया स्पष्ट करें) |  |  |  |

1. **On a scale of “1” to “5” where 5 stands for very satisfied and “1” stands for very dissatisfied, how would you rate your experience at the MHC? SINGLE CODING ONLY**

**1 – 5 के स्केल पर आप अपने एमएचसी के अनुभव को किस प्रकार रेट करेंगे? जहा “5” का मतलब है “बहुत संतुष्ट” और “1” का मतलब “बहुत असंतुष्ट”**

| **1** | **2** | **3** | **4** | **5** |
| --- | --- | --- | --- | --- |
| Very Dissatisfied  बहुत असंतुष्ट | Dissatisfied  असंतुष्ट | Neither satisfied nor dissatisfied  ना संतुष्ट ना असंतुष्ट | Satisfied  संतुष्ट | Very Satisfied  बहुत संतुष्ट |

1. **What suggestions would you give for improvement of services at MHC?**

**एमएचसी में सुधार के लिए आप क्या सुझाव देना चाहेंगे?**

**OPEN ENDED, BUT IF THE RESPONDENT DOES NOT GIVE ANY SUGGESTION CODE “99”**

| Suggestion -1  सुझाव – 1 |  |
| --- | --- |
| Suggestion -2  सुझाव – 2 |  |
| Suggestion -3  सुझाव – 3 |  |
| Suggestion -4  सुझाव – 4 |  |
| No Reason at all  कोई कारण नहीं है | **99** |

**Thank and close the interview.**
